# Acceptability of predictive testing for ischemic heart disease in those with a family history and the impact of results on behavioural intention and behaviour change: a systematic review

**DOI:** 10.1186/s12889-022-14116-6

**Published:** 2022-09-15

**Authors:** Imogen Wells, Gwenda Simons, Clare Davenport, Christian D. Mallen, Karim Raza, Marie Falahee

**Affiliations:** 1 Rheumatology Research Group, Institute of Inflammation and Ageing, College of Medical and Dental Sciences, University of Birmingham, Birmingham, UK; 2 Institute of Applied Health Research, College of Medical and Dental Sciences, University of Birmingham, Birmingham, UK; 3 NIHR Birmingham Biomedical Research Centre, University Hospitals Birmingham NHS Foundation Trust and University of Birmingham, Birmingham, UK; 4 Primary Care Centre Versus Arthritis, School of Medicine, David Weatherall Building, Keele University, Keele, UK; 5 Sandwell and West Birmingham NHS trust, Birmingham, UK; 6 MRC Versus Arthritis Centre for Musculoskeletal Ageing Research and the Research into Inflammatory Arthritis Centre, Versus Arthritis, University of Birmingham, Birmingham, UK

**Keywords:** Ischemic heart disease, Predictive testing, Health behaviour, First degree relatives, Systematic review

## Abstract

**Background:**

Tests to predict the development of chronic diseases in those with a family history of the disease are becoming increasingly available and can identify those who may benefit most from preventive interventions. It is important to understand the acceptability of these predictive approaches to inform the development of tools to support decision making. Whilst data are lacking for many diseases, data are available for ischemic heart disease (IHD). Therefore, this study investigates the willingness of those with a family history of IHD to take a predictive test, and the effect of the test results on risk-related behaviours.

**Method:**

Medline, EMBASE, PsycINFO, LILACS and grey literature were searched. Primary research, including adult participants with a family history of IHD, and assessing a predictive test were included. Qualitative and quantitative outcomes measuring willingness to take a predictive test and the effect of test results on risk-related behaviours were also included. Data concerning study aims, participants, design, predictive test, intervention and findings were extracted. Study quality was assessed using the Standard Quality Assessment Criteria for Evaluating Research Papers from a Variety of Fields and a narrative synthesis undertaken.

**Results:**

Five quantitative and two qualitative studies were included. These were conducted in the Netherlands (*n* = 1), Australia (*n =* 1), USA (*n =* 1) and the UK (*n =* 4). Methodological quality ranged from moderate to good. Three studies found that most relatives were willing to take a predictive test, reporting family history (*n =* 2) and general practitioner (GP) recommendation (*n =* 1) as determinants of interest. Studies assessing the effect of test results on behavioural intentions (*n =* 2) found increased intentions to engage in physical activity and smoking cessation, but not healthy eating in those at increased risk of developing IHD. In studies examining actual behaviour change (*n =* 2) most participants reported engaging in at least one preventive behaviour, particularly medication adherence.

**Conclusion:**

The results suggests that predictive approaches are acceptable to those with a family history of IHD and have a positive impact on health behaviours. Further studies are needed to provide a comprehensive understanding of predictive approaches in IHD and other chronic conditions.

**Supplementary Information:**

The online version contains supplementary material available at 10.1186/s12889-022-14116-6.

## Introduction

Healthcare services are moving away from a ‘one size fits all’ approach to an era of personalised medicine, with a focus on early intervention and disease prevention [1]. There is growing evidence of the efficacy of pharmacological interventions to prevent or delay the onset of a range of chronic diseases and cancers, including ischemic heart disease (IHD) [2, 3], rheumatoid arthritis (RA) [4], diabetes mellitus (DM) [5], and breast cancer [6]. Lifestyle interventions, such as increased physical activity and an improved diet, have also been found to delay or reduce the risk of IHD, DM and breast cancer [2, 7–9]. For IHD and RA, smoking cessation is likely to reduce disease risk [10, 11]. An increasing focus on preventive approaches for chronic diseases increases the need for effective identification of those at risk [12–14]. The presence of a positive family history of the disease of interest (i.e., someone who has a first degree relative (FDR), second degree relative (SDR) etc. with, for example, IHD, DM or RA) is an important and widely understood determinant which can be used to identify a cohort of individuals at increased risk of that disease [15–17]. Specific tests can then be applied to the cohort to identify subgroups with particularly high risk who may benefit the most from preventive approaches [18–20].

Unlike some other chronic conditions, IHD has risk factors, such as family history, smoking, body mass index (BMI) and blood pressure, that are routinely assessed in clinical care and can be incorporated into risk calculators to predict the likelihood of developing future disease [21–25]. Interventions to reduce the risk of IHD can also be integrated into routine clinical care [26–34].

Increasingly precise risk assessments are likely to become available as a result of technological advancements. For example, data from genetic analysis and imaging studies are likely to be incorporated into existing disease prediction algorithms. Predictors that extend beyond conventional assessment for IHD are currently being explored, including genetic testing and blood flow parameters assessed by imaging [35–38]. For example, the use of a gene expression score which measures the expression of 23 genes in peripheral blood has been found to provide enhanced predictive accuracy compared with standard clinical assessment for IHD [38].

With the growth of predictive tools that extend beyond risk factors assessed as part of standard physical examination, such as blood pressure, BMI, or smoking, it is increasingly important to explore their acceptability for those with a family history of IHD, and whether the use of these tools have a positive impact on health behaviours. Exploring this could identify potential barriers and facilitators to the acceptability of risk prediction and inform the development of information and resources to support shared decision making for those considering predictive tests, treatment to reduce risk or taking part in prevention research. Importantly, this information could also usefully inform the development of similar strategies for other multifactorial diseases, such as RA, where risk assessment of asymptomatic individuals with a family history is not integrated into current care but research interest in predictive and preventive strategies is increasing, and there is limited knowledge about the views of at-risk individuals about predictive testing [39–44].

Three systematic reviews of studies of interest in predictive testing for IHD, and other chronic diseases were identified as part of a scoping search for this review. A review of 11 qualitative studies assessing DM, cardiovascular disease (CVD) and inflammatory bowel disease (IBD) published between 1989 and 2015 found that study participants believed predictive testing to be effective at quantifying risk, but some highlighted concerns relating to confidentiality of risk information [44]. That review did not search for potentially relevant studies from the grey literature. Eight of the studies that were included were considered robust, while three were reported to have minor methodological issues. A systematic review of eight observational and experimental studies focusing on DM, CVD and obesity with a search end date of 2012 found a high level of public interest in predictive testing for these diseases, but the included studies only addressed hypothetical predictive tests [45]. Ratings of the methodological quality of the included studies were judged to be positive for six studies, and neutral for two. A systematic review of 13 randomised controlled trials (RCTs) (2003–2015) that assessed DM, CVD and obesity found no consistent effect of predictive testing on intention to engage in risk-reduction behaviours (diet and physical activity) or actual behaviour change [46]. Five studies in that review were judged as having a low risk of bias, four as having unclear risk, and four were judged to have a high risk of bias.

We did not identify any systematic reviews which had focussed exclusively on perceptions of predictive testing for IHD, and thus the findings for individual conditions may be confounded. For example, different outcomes relating to perceived risk or behaviour change may be relevant, and risk assessment tools that are available and/ or routinely offered for each condition may vary. We also did not identify any review in this context that focussed specifically on the perceptions of predictive testing held by individuals who are at risk due to having a family history, or the impact of the test on risk-reducing behaviour for this at-risk group. The current systematic review will therefore address the willingness of those with a family history of IHD to accept a test to predict their risk of developing IHD (that extends beyond risk factors assessed in standard clinical assessment including history and physical examination), and the effect of such testing on intentions to change risk related behaviours or actual behaviour change for this group.

## Method

This review was carried out in accordance with the Preferred Reporting Items for Systematic Reviews and Meta-Analyses (PRISMA) recommendations [47]. The protocol for this review was registered with the University of York, Centre for Reviews and Dissemination (CRD) International Prospective Register of Systematic Reviews (PROSPERO) database: CRD42019124524.

### Search strategy

The search strategy for this review was generated with support from a systematic review expert (CD) and informed by search strategies used in previous related reviews [45, 46]. The search was limited to publications involving adult participants aged 18 and over. The search strategy specified no start date, and the end date was 18th of May 2022. The electronic databases searched were OVID MEDLINE, psycINFO EMBASE and LILACS. The search strategy was designed to be broad enough to efficiently capture literature that was relevant to both research questions. Terms relating to or describing the population, disease and intervention were investigated. Both keywords and medical subject headings were included and adapted for use in each of the bibliographic databases searched. Grey literature was also searched using Google, EThOS and ProQuest, and references from review papers identified in scoping searches and those from studies included in the present review were checked for relevance to the current objectives [45, 46]. The search terms used for each source are provided in an additional word file (see Additional file 1). Database searches were not restricted to a particular language. For LILACS search terms were entered both in English and in Spanish (see Additional file 1).

### Eligibility criteria

In order to be eligible for review, studies identified by the search strategy above had to meet each of the following criteria:

#### Type of study

Any primary research was eligible for review. This included both quantitative and qualitative studies. Systematic reviews were excluded but their included studies were eligible for inclusion. Thesis manuscripts were also excluded but published work deriving from the thesis was eligible for inclusion.

#### Type of participants

Eligible participants were adults (aged 18 or over) with a family history of IHD (defined as heart problems caused by narrowed coronary arteries that supply blood to the heart [48]). Studies including both participants with and without a family history of IHD were eligible for inclusion, provided that results were presented separately.

#### Type of intervention

Eligible studies assessed a predictive test for IHD, defined as a test that can provide information about the likelihood that a person will develop IHD in the future. The information provided by such a test should be additional to that provided by standard physical examination (defined as examination of IHD risk using blood pressure, weight and BMI). The test should involve additional investigation, including but not restricted to, blood tests (to assess genetic variants or cholesterol levels), saliva tests, electrocardiograms (ECGs) and imaging as appropriate. Tests could be actual or hypothetical.

#### Outcome measures

Both quantitative and qualitative outcomes were included. Outcomes of interest were willingness to take a predictive test and the effect of predictive test results on health behaviour, behavioural intentions or clinical outcomes.

Willingness to take a predictive test could be measured by self-reported interest, test uptake or attitudes (positive or negative) towards predictive testing.

A range of health behaviours, behavioural intentions and associated clinical outcomes could be measured to examine the effect of predictive test results. These include, but are not limited to smoking cessation, dietary modification, physical activity modification, treatment/ medication adherence (for example the use of statins), weight loss and changes in serum lipid profile.

### Study selection

Titles and abstracts of studies identified by the search strategy were screened by one of two reviewers (either IW or GS). Both reviewers further screened an overlap of 12% of all sources to assess agreement. When no English abstract was supplied, Google Translate was used and translated abstracts were screened independently by two reviewers (IW and GS). Of the 847 titles and abstracts screened by both reviewers, one or both reviewers were unsure about the inclusion of 15 sources. This was either resolved during discussion between the two reviewers and where no agreement could be reached (*N* = 1) a 3rd reviewer (MF) screened the abstract as well. If studies were deemed potentially eligible at this stage, or where there was any uncertainty about eligibility, they were subject to a full-text review. All full texts were reviewed independently by both IW and MF or GS. Uncertainty occurred over the eligibility of 3 of the 27 full texts reviewed. These discrepancies were discussed and resolved with an additional reviewer (KR).

### Patient research partner input

The review objectives and search strategy were informed by discussion with patient research partners (defined as patients with a lived experience of the disease under investigation who are actively involved in the design/delivery/dissemination of data from research projects). A group of three patient research partners contributed to the analysis and interpretation of findings for this review. As a result of their input, additional demographic data (age, sex, education levels, socioeconomic status (SES) and ethnicity) were extracted from each study, if reported. The impact of these demographic variables on willingness to take a predictive test for IHD and the effect of such testing on health behaviours was assessed.

### Data collection and items

Data for all included papers were assessed and extracted in duplicate between three reviewers (IW, GM and NW) in accordance with the items outlined in Table 1. Discrepancies were discussed with two other authors (MF, KR).

**Table 1:** Data items that were extracted across included studies

Items of study	Data items extracted
Background	Aim, source of funding and ethical approval.
Method	Study design and setting, sample size, participant characteristics (including demographic data), defined family history, patient and public involvement, intervention(s) and predictive test(s) used.
Results	Any quantitative or qualitative outcome measuring willingness to take a predictive test and the effect of test results on risk reducing behaviours and subsequent outcomes, including but not restricted to smoking cessation, dietary modification, physical activity modification, treatment/medication adherence, weight loss and serum lipid profile.

### Risk of bias assessment

The quality of each study was assessed in duplicate between three reviewers (IW, GM, AB) using the Standard Quality Assessment Criteria for Evaluating Research Papers from a Variety of Fields [49]. This validated tool uses a 14-item checklist to evaluate the quality of quantitative studies relating to the reporting of study methods (description of objectives, recruitment, allocation, outcome measures, sampling size and strategy) and results (description of analytic methods, confounding and detail of results). A separate, 10- item checklist was used to evaluate qualitative studies relating to the reporting of study methods (description of objectives, study context, sampling strategy and data collection methods) and results (description of analysis, verification procedures, conclusions and reflexivity). Each study was scored based on the degree to which specific criteria were met (Yes = 2, Partial = 1, No = 0). Items that were not applicable to a particular study design in the quantitative checklist were marked N/A and were excluded when calculating the total score. Assigning N/A was not permitted for any of the items in the qualitative checklist. Any study that had a total score ≥ 75% of the maximum possible score was judged as having good quality, scores between 55 and 75% indicated moderate quality and scores below 55% indicated poor quality [49, 50]. Due to heterogeneity in study designs, the quality indicators for each study type are not directly comparable. However, an overall assessment score can be used as a guide for interpreting the relative and overall quality of evidence from individual studies. Inter-rater agreement was high between researchers (97% agreement for quantitative studies; 92% agreement for qualitative studies). Disagreement between assessors was resolved through discussion amongst the research team. Quality scores were summarised across studies.

### Data synthesis

A narrative synthesis was used to synthesise the findings across all studies included within this review [51]. This approach has been widely used in mixed-method systematic reviews [52, 53], and is particularly useful when synthesising findings in which the review objectives dictate the inclusion of a wide variety of research designs [54]. Quantitative and qualitative data were integrated based on guidance by Popay and colleagues [51, 55]. A framework analysis was conducted, where outcomes from quantitative studies that were relevant to the objectives of this systematic review were used to develop a framework. Concepts from qualitative studies were then synthesised using this framework, and any additional concepts were added as necessary. Similarities and differences between and within each study contributing to a specific theme were then assessed and discussed.

## Results

### Study selection

**Fig. 1:**
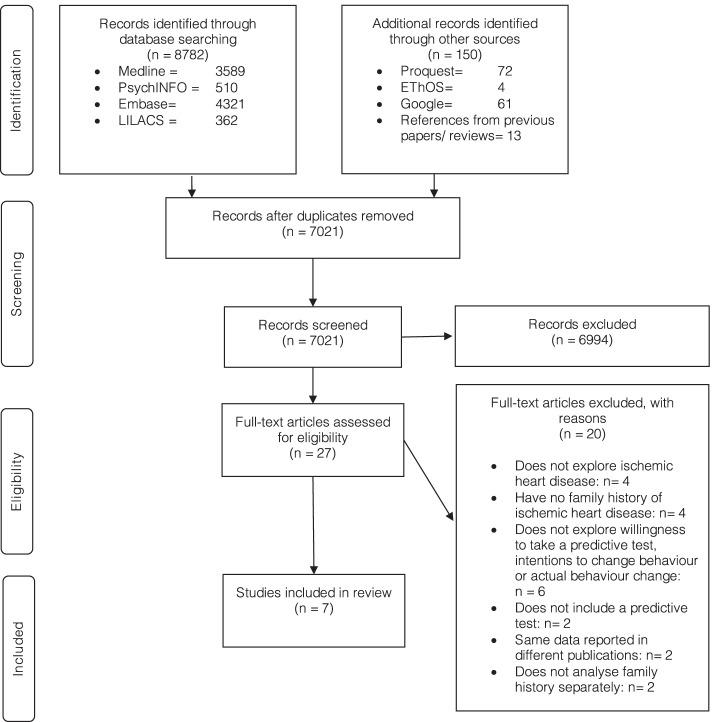
PRISMA flow diagram of the selection process of included studies

Of the 8922 papers identified across all databases, 7021 were screened after deduplication. This resulted in 27 full-text papers being considered, of which seven were included in the review. One of these seven studies identified from the database search was also identified in the reference list of a previous review used to inform the search strategy, and two of the seven included studies were also identified from an included study [45, 56]. Reasons for exclusion of 20 studies are provided in Fig. 1.

### Characteristics of studies

Of the seven studies identified, five employed a quantitative design (two observational, one experimental pre-post-test, and two RCTs), and the remaining two employed a qualitative design (one employed individual interviews and the other utilised individual and couple interviews). Studies were published between 2004 and 2016 and were conducted in the Netherlands (*n* = 1), Australia (*n =* 1), USA (*n =* 1) and the UK (*n =* 4). Study settings included primary care practices (*n =* 2), tertiary care cardiovascular wards (n = 1), university campuses (*n =* 2), and participants’ homes (*n =* 2). The proportion of participants at risk due to a family history of IHD ranged from 22 to 100% across studies, with the average being 65%. From the data reported in these studies, most study participants were between 40 and 65 years of age, 28–87% were female, 21–47% had low levels of education, 24–52% had intermediate levels of education, 20–47% had high levels of education, and 67–97% were of a white ethnicity. Two studies included participants as young as 16 years of age [57, 58]. Whilst this challenges the exclusion criteria, the mean age for participants in the study by Sanderson et al. [57] was 47 (*SD* = 18.2) years, and for the Sanderson and Michie [58] study, participants’ mean age was 34 (*SD* = 12) years for the genetic test-high risk study group, 30 (*SD* = 12) years for the genetic test-low risk group, and 30 (*SD* = 10) years for the oxidative test-high risk group. As a limited number of studies were identified as eligible for inclusion in this review, these studies were included. The number of participants under 18 years of age was not reported in either study, and it was thus not possible to extract data for participants over 18 years of age only. Two studies examined predictive genetic tests, three examined predictive cholesterol tests and two examined both. Willingness to take a predictive test was assessed by three studies. Four studies explored the effect of predictive test results on health behaviours (two investigated behavioural intentions, and two explored self-reported adoption of health behaviours). No studies examined actual health behaviours. The preventive behaviours examined in these studies were physical activity, dietary intake, medication adherence and smoking cessation. All four studies included an intervention informing participants of preventive treatment options alongside risk results.

Table 2 describes the aims, participants, design and setting, type of predictive test, intervention, and findings of each of the included studies. Additional study characteristics are provided in an additional word file (see additional file 2).

**Table 2:** Characteristics of studies included in this review

Reference	Aims	Population	Demographic characteristics	Study design and setting	Intervention and Predictive test	Findings
Claassen et al [59] Netherlands 2012	To examine differences in self-reported perceived risk, causal attributions of IHD^a^, perceived efficacy of preventive behaviour and adoption of preventive behaviour between people with and without a known genetic predisposition of IHD. **Hypotheses:** Those with a known genetic predisposition to IHD compared to those without will: -Have higher perceptions of IHD risk. -Attribute IHD more strongly to genetics and less to an unhealthy lifestyle. -Have more confidence in the efficacy of medication, and less confidence in efficacy of a healthy lifestyle to reduce IHD risk. -No hypotheses for the adoption of preventive behaviour.	100 participants: *n =* 51 individuals with a GP^b^ to IHD, who had a recent diagnosis of familial hypercholesterolemia through DNA testing in a national family cascade screening program in the Netherlands. 15 had one FDR^d^, and 28 had two or more FDRs. *n =* 49 individuals with NGP^c^ but still at risk for IHD. These participants were from a larger interventional study. 16 had one FDR and 12 had 2 or more FDRs.	**Age (mean (SD))-** GP *n =* 54 (13). NGP *n =* 55(8). **Sex-** GP-female *n =* 27 male *n =* 24. NGP- female *n =* 23. male *n =* 26. **Education-** GP- low *n =* 19, medium *n =* 15, high *n =* 16. NGP- low *n =* 23, medium *n =* 14, high *n =* 10.	**Design-** Cross-sectional postal survey that measured self-reported cholesterol levels, blood pressure, number of FDRs with IHD, perceived risk (susceptibility and comparative risk within the next 10 years), causal attributions of IHD (genetic e.g. hereditary/ predisposition and lifestyle e.g. unhealthy diet/lack of exercise/smoking), perceived efficacy of preventive behaviours (medication use for those who were prescribed medication, dietary behaviour, exercise and smoking cessation) and reported preventive behaviour (medication adherence, diet, exercise and smoking). **Setting-** Netherlands.	**Intervention-** The study directly compared two different populations who experienced different types of risk assessments (genetic and cholesterol). **Predictive test(s)-** DNA test (for those in the genetic predisposition condition) and blood test to measure cholesterol levels.	Perceived comparative risk, genetic attributions to developing IHD and perceived efficacy of taking medication was significantly higher in those who had a genetic compared to a cholesterol test (28% higher for perceived comparative risk (*p =* 0.003), 11% higher for genetic attributions (*p =* 0.02) and 16% higher for perceived efficacy of taking medication (*p =* 0.001)).No differences between groups in terms of perceived susceptibility of IHD, lifestyle attributions for developing IHD, perceived efficacy of a healthy lifestyle or preventive behaviour. Those with a higher number of FDRs with IHD reported higher perceived susceptibility to IHD (*p =* 0.04), stronger genetic attributions (*p =* 0.03), increased confidence in the efficacy of medication (*p =* 0.04) and reported engaging in physical activity and a healthy diet more often than those with a lower number of FDRs (*p =* 0.005). Medication adherence was high for those who took a genetic or cholesterol test (96 and 97%, respectively), and did not differ based on family history. The number of participants who reported not smoking was high for those who took a genetic or cholesterol test (88 and 82%, respectively), and did not differ based on family history.
Imes et al [60] USA 2016	To examine the effect of a pilot intervention for young adults with a family history of IHD on IHD knowledge, perceived IHD risk, and intention to engage in IHD risk reducing behaviours. **Hypothesis:** Perceived IHD risk, IHD knowledge and IHD risk reducing behaviours will increase from baseline to post-intervention.	*n =* 15 undergraduate and postgraduate students. 1 participant had an FDR with IHD, and 12 had at least one SDR^e^ with IHD.	**Age (mean (SD))** 20.8 (2.2). **Sex-** Female *n =* 13. Male *n =* 2. **Ethnicity-** Caucasian *n =* 10. Asian *n =* 2. Asian and Native Hawaiian *n =* 1 Hispanic or Latino *n =* 2. Black *n =* 0.	**Design-**Experimental pre-post test pilot study. A self-report questionnaire measured IHD knowledge, perceived IHD risk and intention to engage in IHD risk reducing behaviours (diet and physical activity) at baseline and 2 weeks post-intervention. **Setting-** Pacific Northwest University, Washington, USA.	**Intervention-** IHD risk assessment incorporating information on family history (three-generation pedigree), a blood test to assess lipid levels, and a brief educational counselling intervention on increasing physical activity and dietary behaviours. **Predictive test-** Blood test to assess lipid levels associated with elevated IHD risk (LDL-C, HDL-C and triglyceride levels).	Participants’ IHD knowledge significantly increased post-intervention compared to baseline (95% CI of the difference 0.80–9.01, *p =* 0.02). Participants’ perceived risk of IHD was significantly higher post-intervention compared to baseline (Z = 1.97 (95% CI 0.02–1.98), *p =* 0.048). However, when adjusting for multiple comparisons (perceived lifetime risk, perceived susceptibility, and perceived lifetime risk after receiving family history information only) this effect was no longer significant. Participants expressed a higher intention to engage in exercise after receiving the intervention compared to baseline (Z = 2.09 (95% CI 0.36–1.28), *p =* 0.036), with a medium effect size (*d =* 0.58). However, after adjustment for multiple comparisons (engagement in exercise, diet, health-promoting lifestyles (total score for exercise and diet), and likelihood to engage in a health-promoting lifestyle after receiving family history information only, *p* ≤ 0.0125) this effect was no longer statistically significant. Participants’ intention to adopt a healthy diet did not differ from baseline to post-intervention (Z = 1.85 (95% CI − 0.04-0.53), *p =* 0.064). Those with a higher number of first- and second-degree relatives with IHD had significantly higher risk perceptions of IHD (*p =* 0.014), and a significantly higher intention to exercise after receiving the intervention than those with a lower number of first- and second-degree relatives (*p =* 0.035). This result was not found for diet.
Middlemass et al^1^ [56] UK 2014	To explore how patients who had a recent IHD assessment perceive additional information from genetic testing for IHD, and perceptions of whether this additional genetic information influenced their behaviour.	*n* = 29 individuals from primary care practices who were part of a larger ongoing study and who had received a conventional IHD risk assessment within 18 months prior to this study and had agreed to have a genetic test. 17 had either an FDR or SDR with IHD.	**Age (median (IQR))-** 59 (53.5–62). **Sex-** Female *n =* 8. Male *n =* 21. **Ethnicity-** Caucasian *n =* 28. Asian *n =* 0. Mediterranean *n =* 1. Black *n =* 0. **Education-** No formal qualifications *n =* 6. GCSE *n =* 2. Vocational qualification *n =* 3. A-level *n =* 2. First degree *n =* 10. Other *n =* 5. Missing *n =* 1	**Design-** Qualitative interview study. Interviews were conducted 4 months after receiving risk results (conventional, genetic and overall risk, communicated by letter). Participants were asked about their conventional risk assessment, experience of the genetic test, their interpretations of the genetic and conventional risk results, and whether the results had influenced any change in their behaviours. **Setting-** 12 primary care practices from both urban and rural settings in Nottinghamshire, UK.	**Intervention-** There was no intervention for this study, as all participants had a genetic (and conventional) test. **Predictive test-** Saliva sample which involved an IHD panel of nine risk alleles to produce a combined risk profile score. For the conventional IHD risk assessment conducted previously, a blood sample was taken to measure cholesterol levels.	Family history was cited as the primary motivation for having a genetic test, so they can clarify their family history further and are able to discuss their results with their children. Testing was seen as beneficial as it could motivate behaviour change, particularly in those with high genetic and conventional risk results. However, for some individuals identified as at high risk from a conventional IHD risk assessment, an average genetic risk score provided false reassurance that they did not have to modify their lifestyle to reduce their risk. Genetic testing was cited as being more appropriate for a younger age group as prevention is more likely to lead to health benefits.
Stocks et al [61] Australia 2015	**Primary aim:** To determine whether the provision of advice promoting IHD risk assessment to FDRs of patients with premature IHD (PIHD) would increase the proportion of relatives who undertake IHD risk assessment. **Secondary aim:** To ascertain absolute IHD risk of relatives in the intervention group.	*n =* 97 FDRs (siblings and children) of patients hospitalised with PIHD in tertiary care cardiovascular wards in South Australia. *n =* 55 were in the intervention group for this study. *n =* 42 were in the control group.	**Age-** 18 and over. **Sex-** Female *n =* 59. Male *n =* 38.	**Design-** Prospective randomised- controlled trial. Patients were randomly allocated to provide an information pack (either intervention or control) to their FDRs either in the hospital or via post. Evidence that relatives had an IHD risk assessment was provided by GPs through a postcard returned to the University, detailing relatives’ risk results. FDRs were also phoned 6 months after providing consent to ascertain whether they had attended their GP for a IHD risk assessment within those 6 months, whether any IHD risk factors were identified and whether any lifestyle changes had been made. **Setting-** Tertiary care cardiovascular wards at Royal Adelaide Hospital, Flinders Medical Centre and Flinders Private Hospital, Australia.	**Intervention-** Written advice/ recommendation to attend their GP for a risk assessment. **Predictive test-**Blood test to measure total cholesterol: HDL ratio.	52% of all FDRs attended their GP for an IHD risk assessment within 6 months of the trial, 75% from the intervention group and 21% from the control group (difference (in proportions) 53% (95% CI 36–71%). More FDRs from the control group compared to the intervention group did not see their GP at all during the 6-month follow-up (41% vs 15%, respectively). A small number of FDRs from the control and intervention groups attended the GP for a risk assessment after 6 months (17 and 2%, respectively). The majority of FDRs from the intervention group who attended their GP had low IHD risk (66%). All FDRs who had moderate to very high IHD risk (34%) were siblings.
Saukko et al^1^ [62] UK 2012	To explore how individuals who are at high risk of heart disease configure risk information provided by an IHD risk assessment, and how their understanding of their risk may shape their preventive behaviours.	*n =* 30 participants who were taking part in a trial assessing the utility of family history in an IHD risk assessment. 20 participants from the current study were in the intervention group in the trial, and 10 were in the control group. Maximum variation sampling was conducted in the current study based on sex, socioeconomic status, and family history. 11 participants in the intervention group had a family history of IHD. The degree of family history was not disclosed. Family history was not assessed in the control group.	**Age (range)-** 30–49 *n =* 2. 50–59 *n =* 11. 60–65 *n =* 17. **Sex-** Female *n =* 10. Male *n =* 20. **Employment-** Managerial and professional *n =* 10. Intermediate *n =* 5. Manual and unemployed *n =* 15.	**Design-** Qualitative interview study, nested within a trial. Participants took part in interviews at 2 weeks and 6 months after receiving risk results. These interviews asked participants what they thought about being classed as at high risk of IHD, their thoughts about the risk assessment, what interaction they’d had with their clinicians about their risk, and what they’d done with the information. **Setting-** 24 general practices in diverse socioeconomic areas in the East Midlands and Southwest of the UK.	**Intervention-**The current study was not an interventional study, but for the nested trial participants were invited to discuss their risk, lifestyle and medications with their clinicians. The intervention arm of the trial had their family history of IHD formally assessed (self-report). **Predictive test-**Blood test to measure cholesterol (conducted as part of the trial).	Initially, most participants were shocked to be identified at high risk of IHD and planned some preventive actions to reduce their risk. At the 6 month follow up, 23 participants reported engaging in health behaviours; 13 reported taking statins only, five reported taking part in lifestyle behaviours (diet and physical activity) and five reported taking statins and engaging in lifestyle behaviours. Seven reported not engaging in any health behaviours, which was often due to overwhelming personal and social circumstances. Participants in this group often had a lower socioeconomic status than those who engaged in risk-reducing behaviours and had poor communication with their clinicians. No substantial difference was found in these results between those in the intervention and control groups of the nested trial, or those with and without a family history.
Sanderson and Michie [58] UK 2007	To investigate the impact of IHD risk test type (genetic high risk, genetic low risk and oxidative high risk) on intention to quit smoking. To examine whether the impact of test type is the same for individuals with a family history compared to without. **Hypotheses:** -Smokers in the genetic high-risk group would have lower outcome expectations about quitting smoking, and lower perceived control over quitting than the oxidative high-risk group. -Outcome expectations and perceived control would partly mediate the effect of test type on intention to quit smoking.	*n =* 261 smokers overall: *n =* 75 were undergraduate students from a UK university. *n =* 161 were undergraduate students’ family/friends or staff from a UK university. *n =* 25 were recruited through Quitline- a telephone quit-smoking service. All participants smoked seven or more cigarettes per week. 58 had a family history of IHD. The degree of history was not disclosed.	**Age (mean (SD))-** Genetic high risk- 34 (12). Genetic low risk- 30 (12). Oxidative high risk- 30(10). **Sex-** Female *n* = 116. Male *n* = 145. **Ethnicity-** Caucasian *n =* 217. Non-Caucasian *n =* 44. **Education-** GCSE *n =* 49. A-level *n =* 88. Bachelor’s degree *n =* 89. Higher degree *n =* 34.	**Design-**Randomised controlled trial. Self-reported family history was measured at baseline, intention to quit smoking was measured at baseline and post-intervention. Self-reported attitudes towards quitting smoking, outcome expectations, perceived control over quitting, and perceived social pressure was measured post-intervention using a questionnaire. **Setting-** UK.	**Intervention-** Hypothetical predictive test result from one of three types of predictive test: high genetic risk vs low genetic risk vs high oxidative stress risk. **Predictive test-** Hypothetical genetic or oxidative stress test, described as a blood test that tests for different genes (genetic risk) or enzymes and antioxidants (oxidative stress) associated with IHD risk.	Those who received a high genetic risk result had significantly higher outcome expectations (*p <* 0.001), more positive attitudes towards quitting smoking (*p =* 0.039) and had a higher intention to quit smoking than those who received a high oxidative risk result (*p =* 0.009). No significant difference was found for perceived control or social pressure. The genetic high-risk group also had significantly higher outcome expectations (*p =* 0.003), greater perceived control over quitting smoking (*p =* 0.012) and a stronger intention to quit (*p <* 0.001) than those who received a genetic low risk result. No significant difference was found for perceived attitudes towards quitting or perceived social pressure. 30.3% of the effect of risk results on intention to quit smoking was mediated by stronger beliefs that quitting smoking would reduce risk of IHD (outcome expectations) (*p =* 0.011). When examining the interaction between family history and risk results, the effect of a high genetic risk result on outcome expectations was greatest amongst smokers with no family history of heart disease. (*p =* 0.04). The interaction between family history and risk results was not significant for attitudes towards quitting (*p =* 0.61), perceived control over quitting (*p =* 0.27), perceived social pressure (*p =* 0.58) or intention to quit (*p =* 0.34).
Sanderson et al [57] UK 2004	To examine interest in genetic testing for IHD and cancer, and the influence of factors such as family history, age, sex, education, and ethnicity on interest. **Hypotheses:** **-**The same factors that predict interest in genetic testing for cancer (previously, younger adults, women and those with a higher level of education) will predict interest in genetic testing for IHD. -People will be more interested in genetic testing for IHD than for cancer.	*n =* 1960 respondents from a stratified random probability sample as part of the Office for National Statistics Omnibus survey. 830 respondents had at least one FDR or SDR with IHD.	**Age (mean (SD))-**47(18.2). **Sex-** Female *n =* 999. Male *n =* 961. **Education-** No formal qualifications *n =* 629. GCSEs *n =* 524. A-levels *n =* 559. Degree *n =* 247. **Ethnicity-** Caucasian *n =* 1843. Non-Caucasian *n =* 112.	**Design-** Cross-sectional survey study. The survey questions were delivered by researchers through telephone calls to participants. Participants were asked if they would take a genetic test for IHD (and cancer) in the next 6 months. They were also asked about their age, sex, ethnicity, education, and family history. **Setting-** UK.	**Predictive test-** Hypothetical genetic test.	Respondents were significantly more likely to be interested in taking a predictive test for IHD (mean 2.95 (95% CI of the difference 2.91:3.01)) than for cancer (mean 2.83 (95% CI 2.78:2.88), *p <* 0.001). 42% of participants would definitely take a predictive test for IHD and 28% would probably take a test. Men were significantly more likely to say they would take a predictive test for IHD than women (OR 0.79 (0.65:0.97)), respectively, *p <* 0.05). Middle-aged respondents (46–60 years) were more interested in predictive testing for IHD than those aged 16–30 years (OR 1.99 (1.45:2.75), p < 0.001). Those aged 31–45 years (OR 1.43 (1.08:1.90)) and > 75 years (OR 0.61 (0.39:0.94)) were also more interested in testing than those aged 16–30 years, *p <* 0.05). Compared to those with a university degree, those with school-based qualifications such as GCSEs (OR 1.90 (1.35:2.66)) and A-levels (OR 1.99 (1.43:2.79)) had a higher interest in testing, *p* < 0.001. No significant difference in interest in testing for IHD was found for ethnicity (*p =* 0.16). Participants who had at least one FDR or SDR with IHD had a significantly higher interest in predictive testing compared to those who did not have a family history (OR 1.36 (1.09:1.66), *p* < 0.01). Those who did not know their family history showed no greater interest in testing than those who did not have a family history of IHD (OR 1.20 (0.59 to 2.43)).

^a^*IHD* ischemic heart disease, ^b^*GP* genetic predisposition, ^c^*NGP* no genetic predisposition, ^d^*FDR* first degree relative, ^e^*SDR* second degree relative. ^1^ = Qualitative studies. Claassen et al. [59] and Sanderson and Michie [58] did not report confidence intervals

### Risk of bias

Individual and total quality scores for each of the included studies are presented in Tables 3 and 4. Total quality across all studies was moderate to good, with scores ranged from 60 to 100%; 79–100% across quantitative studies and 60–85% across qualitative studies. The manuals, including the criteria used to guide quality assessment and generate overall quality scores for both quantitative and qualitative studies are provided as supplementary material (see Additional file 3). Reflexivity in qualitative studies was defined by the criteria as evidence that the researcher has explicitly assessed the likely impact of their own personal characteristics (age, sex, professional status) and the methods used on the data obtained.

**Table 3:** Quality appraisal checklist and total quality score for included quantitative studies

Criteria^**a**^	Claassen et al [59]	Imes et al [60]	Stocks et al [61]	Sanderson and Michie [58]	Sanderson et al [57]
Question / objective sufficiently described?	2	2	2	2	2
Study design evident and appropriate?	2	2	1	2	2
Method of subject/comparison group selection or input variables described and appropriate?	2	2	2	2	2
Subject characteristics sufficiently described?	2	2	2	2	2
If interventional and random allocation was possible, was it described?	N/A	N/A	2	2	N/A
If interventional and blinding of investigators was possible, was it reported?	N/A	N/A	0	0	N/A
If interventional and blinding of subjects was possible, was it reported?	N/A	N/A	1	0	N/A
Outcome and (if applicable) exposure measure(s) well defined and robust to measurement / misclassification bias?	2	2	2	2	2
Sample size appropriate?	0	0	1	2	2
Analytic methods described/justified and appropriate?	2	2	2	2	2
Some estimate of variance is reported for the main results?	1	2	2	1	2
Controlled for confounding?	1	0	1	2	N/A
Results reported in sufficient detail?	2	2	2	2	2
Conclusions supported by the results?	2	2	2	2	2
Total score (%)	82%	82%	79%	82%	100%

^a^Yes = 2, Partial = 1, No = 0, or not applicable (N/A). Summary score calculated as: ((number of yes × 2) + (number of partials ×1))/(28-(number of N/A × 2))

**Table 4:** Quality appraisal checklist and total quality score for included qualitative studies

Criteria^**a**^	Middlemass et al [56]	Saukko et al [62]
Question / objective sufficiently described?	2	1
Study design evident and appropriate?	1	2
Context for the study clear?	1	2
Connection to a theoretical framework / wider body of knowledge?	0	2
Sampling strategy described, relevant and justified, and includes full range of relevant cases?	1	2
Data collection methods clearly described and systematic?	1	2
Data analysis clearly described and systematic?	2	2
Use of verification procedure(s) to establish credibility?	2	2
Conclusions supported by the results?	2	2
Reflexivity of the account?	0	0
Total score (%)	60%	85%

^a^Yes = 2, Partial = 1, No = 0. Summary score calculated as: ((number of yes × 2) + (number of partial ×1))/ 20

### Summary of quality across studies

A range of sampling strategies were used to recruit participants across the five quantitative studies, including stratified random probability sampling (*n =* 1), convenience sampling (*n =* 1) and purposive sampling (*n =* 3. One of these studies selected those from larger, ongoing studies). The majority of studies measured outcomes using self-report data (*n =* 4). In one study, participants’ general practitioners (GPs) reported their outcome (uptake of a predictive test) in addition to participants’ self-report [61]. Three studies were judged to have issues relating to small sample sizes and/or limited generalisability [59–61]. Two studies reported methodological issues. These issues included the employment of a single group design [60], no manipulation checks to determine participants’ understanding of the information provided [58] and the use of a 2 × 1 instead of a 2 × 2 ANCOVA design [58]. The use of a 2 × 2 ANCOVA design would have generated a more rigorous examination of interaction effects.

One of the two qualitative studies used maximum variation sampling to identify participants from an ongoing trial [62], and the other used a self-selected sample from a larger ongoing study [56]. Both studies were rated zero for reflexivity.

The themes identified for each outcome are as follows. For willingness to take a predictive test (3.5), themes included attitudes towards predictive tests (3.5.1) and uptake of predictive tests (3.5.2). For the effect of predictive testing on behaviour change (3.6), themes were based on the type of behaviour examined, for example: physical activity (3.6.1), diet (3.6.2), medication adherence (3.6.3) and smoking cessation (3.6.4). This synthesis was conducted across both quantitative and qualitative research.

### Willingness to take a predictive test

#### Attitudes towards predictive tests

Participants’ attitudes towards taking a predictive test were examined in one quantitative [57] and one qualitative study [56]. In the qualitative study, where all participants accepted genetic testing in addition to having a standard risk assessment previously, those with a family history of IHD (first or second degree relative) reported that genetic information could increase their awareness of their risk, enable them to inform their children of their risk, and was more likely to motivate preventive behaviour change. However, receiving an average genetic risk result provided false reassurance (reassurance that they did not need to take action to reduce their risk) to some individuals who had previously been identified as at high risk from a conventional IHD risk assessment, which included a cholesterol test [56]. Relatives communicated a desire to clarify their risk from their family history further, convey their risk results to their children and protect their children from developing the disease: “*So all I am interested in, in reality, is protecting my kids and myself. And I think through this genetic thing we should be able to do it hopefully”* ^56(p.e284)^. However, some were sceptical of the value of informing their children, suggesting that they were too young to be concerned about IHD, despite the majority of their children being adults. Another participant stated that predictive testing would be most appropriate for a younger age-group, where preventive measures would be more likely to lead to health benefits: *“I think 25 ... At least it would point to them and, er, give them plenty of time to adjust to the lifestyle”* ^56(p.e286)^. Family history was seen as an important motivator for predictive testing (both hypothetical and genuine) across both studies. In the quantitative survey (which assessed interest in a hypothetical genetic test using multivariable logistic regression), those who knew they had an FDR or SDR with IHD had a greater interest in genetic testing compared to those who did not have an FDR or SDR with IHD (OR 1.36 (1.09:1.66), *p* < 0.01). Those who did not know if they had one FDR or SDR with IHD showed no greater interest in genetic testing than those who did not have a FDR or SDR with IHD (OR 1.20 (0.59:2.43)) [57]. This quantitative study also measured the impact of age on interest but across a wider range of age groups and found that middle-aged participants (defined as those aged 46–60 years) were more interested in predictive testing compared to those aged 16–30 (OR 1.99 (1.45:2.75), *p* < 0.001) [57]. Those aged 31–45 (OR 1.43 (1.08:1.90)) and > 75 years (OR 0.61 (0.39:0.94)) were also more interested in genetic testing compared to those aged 16–30, *p* < 0.05. In addition, the study also found that sex and education levels influenced interest in predictive testing for IHD. Males were more interested in predictive testing compared to females ((OR 0.79 (0.65:0.97)), *p* < 0.05) and, compared to those with a university degree, interest in testing was higher for those whose highest level of education was school-based qualifications such as GCSEs (OR 1.90 (1.35: 2.66)) or A-levels (OR 1.99 (1.43:2.79), *p* < 0.001). It should be noted that analysis of the effect of demographic variables on interest in predictive testing in the quantitative study was not conducted separately for those with a family history compared to those without a family history.

#### Uptake of predictive tests

One prospective RCT investigated FDR’s uptake of a blood test to measure cholesterol levels to assess risk of IHD using a generalised linear model [61]. That study explored whether a recommendation to attend a GP for a risk assessment for IHD in addition to receiving standard information about IHD and cardiovascular risk factors compared with receiving standard information alone would increase the number of relatives who would attend for a risk assessment within 6 months. The proportion of relatives who attended their GP for a risk assessment within 6 months of the trial was 75% in the intervention group compared to 21% in the control group (difference (in proportions) 53% (95% CI 36–71%)). All participants in both the intervention and control arms were FDRs of IHD patients.

### Effect of predictive testing on behaviour change

#### Physical activity

The effect of predictive cholesterol test results on intention to engage in physical activity were examined using a pre-post-test experimental design [60]. After being informed of their cholesterol test results alongside information about the degree of their family history and an educational counselling intervention, relatives reported a significantly greater intention to engage in physical activity post-intervention compared to baseline (Z = 2.09 (95% CI 0.36–1.28), *p* < 0.05). However, this was no longer statistically significant after applying a Bonferroni adjustment for multiple comparisons across intentions to adopt different health behaviours. The degree of family history significantly influenced intention to engage in physical activity. Those who had a higher number of FDRs or SDRs with IHD reported a higher intention to engage in physical activity after receiving the intervention than those with a lower number of relatives with IHD (*r* = .55, *p* < 0.05) [60].

Two further studies, one quantitative and one qualitative, explored the influence of predictive test results on self-reported physical activity [59, 62]. The former investigated self-reported physical activity in those who had a predictive genetic test or conventional IHD risk assessment (which included a cholesterol test) and had received an intervention informing them of risk reduction behaviours. That study found no difference in self-reported physical activity between those who had a genetic test and those who had a cholesterol test. Those who had a higher number of FDRs reported engaging in higher levels of physical activity after taking a genetic or cholesterol test and receiving these test results more often than those with a lower number of FDRs [59]. In a qualitative study of participants identified from a cholesterol test as being at high risk of developing IHD (who were interviewed either alone or with their partner) ten out of 30 reported engaging in increased physical activity [62]. The accounts of those with a family history in that study were not substantially different to those without a family history. Participants in that study were invited to discuss their lifestyle and medications with their clinicians prior to interview. Participants said that, over the 6 months period being investigated, they increased their activity levels as they had negative attitudes towards preventive pharmacological interventions and felt that physical activity was more ‘natural’. When a doctor suggested to a participant that he take medication to reduce his cholesterol, he said he was “*not one to pop pills*”^62(p.569)^ and would rather do it “*naturally*” ^62(p.569)^.

#### Diet

A pre-post-test experimental design was used to examine the effect of predictive test results on intentions to adopt a healthy diet [60]. No evidence of an increased intention to adopt a healthy diet after receiving cholesterol test results, alongside information about the degree of family history and an educational counselling intervention was found compared to baseline (Z = 1.85 (95% CI -0.04-0.53), *p* = 0.06). This was not influenced by the number of FDRs or SDRs with IHD [60].

One cross-sectional survey and one qualitative interview study examined the influence of predictive test results on a reported change in dietary behaviour. The cross-sectional survey study [59] found no difference in self-reported dietary behaviour between those who had a genetic test and those who had a cholesterol test. Participants’ degree of family history was more predictive of dietary behaviour than the type of predictive test in that study, as those who had a higher number of FDRs reported eating healthily every day after receiving their test results more often than those with a lower number of FDRs [59]. The qualitative interview study found that 10 out of 30 participants reported adopting a healthy diet after receiving cholesterol test results identifying them as at high risk for developing IHD. The accounts of participants in this study did not substantially differ between those who had a family history and those who did not [62]. Those who reported adopting a healthy diet after receiving their test results did so because they felt it was more ‘natural’ than preventive medication. Those who reported not adopting a healthy diet after finding out their risk attributed this to their confusion regarding the effectiveness of dietary change for reduction of IHD risk. Participants felt that inconsistent information had been presented to them by various sources, including healthcare professionals: ‘“*We’ve got one book that says you can eat eggs and another book that says you can’t eat eggs*” ^62(p.566)^. One participant added that in the list healthcare professionals gave him about foods to eat “*there was nothing there that you can grasp hold of*” ^62(p.566)^.

#### Medication adherence

One cross-sectional survey study and one qualitative interview study explored the influence of the results of predictive testing on reported medication adherence. The cross-sectional study found no difference in reported medication adherence (to statins or anti-hypertensives) between those who had a genetic test compared with those who had a cholesterol test. Reported medication adherence was exceptionally high in both groups (96 and 97%, respectively) [59]. This was not influenced by the number of FDRs with IHD. In the qualitative interview study, the majority of participants (18 out of 30) also reported adhering to prescribed statins after receiving cholesterol test results. This medication was prescribed once they received their risk results. Participants’ accounts did not substantially differ in those with or without a family history [62]. Factors motivating adherence were varied, with some reporting that they had tried engaging in lifestyle-related behaviours, such as diet and physical activity, but were informed by a healthcare professional that this alone did not lower their risk of IHD. Instead, healthcare professionals cited that statins were a more effective way of lowering risk. For example, a participant reported that a nurse had mentioned *“you can eat the best diet and [be] best weight and God knows what, but you won’t bring your cholesterol down. You’ve got to have tablets”* ^*62(p.566)*^*.* This meant that some participants felt they had no behavioural control over their risk of IHD, and so drug treatment was felt to be necessary. Participants who did not report adhering to taking medication in this study, or any other risk-reducing behaviours generally had lower SES [61]. Those with lower SES reported having poor communication with their clinicians which often left them confused about preventive treatment. One such participant mentioned that she was dissatisfied with doctors, who kept *“pooh poohing”* ^*62(p.571)*^ her and made her feel like she was *“a bit of a waste of space”* ^*62(p.571)*^ when she asked them to take her blood pressure.

#### Smoking cessation

One RCT investigated the effect of the type of predictive test result on intention to stop smoking. Only 22% of participants in this study had a family history of IHD. Participants were provided with hypothetical test results and information about how smoking cessation can reduce IHD risk [58]. Participants were randomly assigned to a genetic test scenario, where they received either a high or low risk result, or an oxidative stress test scenario, where they received a high-risk result. Those who received a genetic risk result indicating that their risk of developing IHD was high had a greater intention to stop smoking than groups presented with a low genetic risk result (3.71 vs 2.98, *p* < 0.001). Additionally, those who received a high genetic risk result had a greater intention to stop smoking than groups presented with a high oxidative risk result (3.71 vs 3.29, *p* < 0.05). This effect did not differ between participants with FDRs or SDRs with IHD and those without (*p =* 0.34). However, 30 % of the effect of test type (genetic or oxidative stress) on intention was mediated by stronger beliefs that stopping smoking would reduce their chance of developing IHD (outcome expectations) and this effect was greatest among those with no first- or second-degree relatives with of IHD, compared to those with first- or second-degree relatives (*p* < 0.05). Therefore, while a genetic high-risk result significantly increased intention to stop smoking in those with a family history, this effect was not as strongly influenced by outcome expectations as those without a family history.

One cross-sectional survey study explored the effect of predictive test results on reported smoking behaviour [59]. That study found no difference in smoking cessation between those who had a genetic test compared with those who had a cholesterol test, or between those who had more or fewer FDRs with IHD. A relatively high number of participants reported not smoking across both groups (88% of those who had a genetic test and 82% of those who had a cholesterol test).

## Discussion

This review has summarised the literature on willingness to take a predictive test in those with a family history of IHD and the effect of results of such tests on approaches to risk-reducing interventions. It is the first review to focus exclusively on studies assessing individuals with a family history of IHD.

Only three studies explored attitudes towards predictive testing or uptake of predictive testing, highlighting the limited evidence available in this area. The evidence available suggests that participants’ degree of family history may be an important determinant of willingness to take a predictive test but further good quality research in this area is needed across those who are at risk due to their family history to provide a comprehensive account.

The relationship between willingness to take a predictive test and family history aligns with literature for other chronic diseases such as breast and ovarian cancer, where the opportunity to inform children and the potential for early treatment intervention are key motivators for acceptance of predictive testing [63]. The influence of family history has been identified across both quantitative and qualitative studies and various diseases [56, 57, 63], suggesting that risk status due to family history is likely to be important to support decision-making around taking a predictive test.

The type of test may also be important in decision making, as one included study suggests relatives placed more value on genetic rather than cholesterol test results as an influence on their behaviour [56]. Genetic tests do not form part of standard clinical care for IHD, whereas cholesterol tests are widely used in this context, and thus the former may be perceived as having added value. Further investigation of the impact of different types of tests, and the integration of predictive information from biomarkers is needed for IHD and other chronic diseases.

Evidence from one included study suggested that interventions recommending predictive testing promoted uptake [61], however, further research is needed on the effectiveness of interventions to promote testing, to inform shared decision making.

In this review, age was observed to influence willingness to take a predictive test [57], although the conclusions that can be drawn from this finding specifically for individuals with a family history of IHD are limited, as no included study examined the effects of individuals’ age on their willingness to take a test separately for those with and those without a family history. This highlights the need for further research exploring the influence of demographic variables on willingness to take a predictive test for those at risk of the condition.

The limited evidence examining the effect of predictive tests on risk-reducing behaviours reported a positive impact of predictive testing on behavioural intentions or self-reported behaviour change. However, no studies assessed the impact of predictive testing on independently observed behavioural change.

After receiving genetic or cholesterol test results and information about preventive behaviours, higher perceived risk (through family history or personal genetic risk, identified by a positive test result) increased physical activity and smoking cessation intentions [58, 60]. Additionally, the majority of participants reported engaging in at least one preventive behaviour, particularly medication adherence [59, 62]. This may be because medication adherence requires less effort compared to lifestyle change and was promoted by healthcare professionals. The type of predictive test (a genetic or cholesterol test) did not appear to influence reported behaviour change. However, other factors did appear to influence participants’ reported physical activity and dietary behaviours, which varied across study designs. This includes the degree of family history in the quantitative study [59], and participants’ preferences for certain behaviours in the qualitative study [62].

Studies exploring other chronic diseases such as RA and DM have found mixed results for the effect of the provision of information about personal risk status on behavioural intentions, as higher personal risk increased intentions to engage in dietary change, physical activity and smoking cessation for some yet had no effect on intention for others [42, 43, 64]. Further research in this area could usefully shed light on the variation of behavioural intentions from increased personal risk across chronic diseases. Studies exploring reported behaviour change across multiple chronic conditions including DM and obesity in healthy participants or those at risk due to clinical characteristics such as raised BMI, found mixed results for the effect of predictive genetic test results on reported lifestyle behaviours [65–68]. The effect of predictive test results on reported behaviour change may differ across chronic diseases, which may be attributable to the perceived severity of a disease [69, 70]. Further studies are needed to investigate the relationship between illness perceptions and engagement with predictive and preventive strategies across chronic diseases.

### Strengths and limitations

This review has several methodological strengths, including a comprehensive search strategy, multidisciplinary contributors, patient partner involvement, and independent assessment for the inclusion of studies, data extraction and quality assessment.

The evidence available for inclusion in this review was limited in its extent - only a small number of studies focused on those with a family history.

Some of these only included a small proportion of participants with a family history, and for one study the total number of those with a family history could not be established [62]. Furthermore, the degree of family history was not fully defined within some studies, for example a distinction between first- and second-degree relatives was not always made.

### Implications

The current review highlights opportunities for further research both for IHD and for other chronic diseases where predictive testing for those at risk due to a family history may be useful, such as RA and DM. Currently, only a few qualitative studies have explored perceptions of predictive testing for these diseases in those at risk [39–44]. Therefore, understanding of predictors of interest in predictive testing is limited.

The findings of this review are informative for the development of interventions to support decision making around taking a predictive test for IHD and other chronic diseases where prevention is possible.

## Conclusions

Since the majority of responses from participants in the studies included in this review indicated a willingness to take a predictive test and to adopt preventive behaviours, evidence from this review suggests that first and second-degree relatives were willing to take a predictive test and reported willingness to adopt preventive behaviours. This was primarily motivated by increased perceived risk of IHD (through family history or personal risk from a positive test result), or a preference for engaging in a certain type of behaviour. However, few studies were identified, highlighting a need for further research to provide more robust evidence to inform strategies to support decision-making in individuals considering a predictive test or preventive intervention for IHD, as well as other chronic diseases where prevention is possible.

## Supplementary Information

**Additional file 1.** Search strategies from each database used in this review.

**Additional file 2.** Table of additional information extracted from included studies.

**Additional file 3.** Manuals used to guide quality assessment and generate overall scores for quantitative and qualitative studies.

## Data Availability

All data generated or analysed during this study are included in this article (and its supplementary information files).

## Abbreviations

IHD: Ischemic heart disease
RA: Rheumatoid arthritis
DM: Diabetes mellitus
FDR: First degree relative
SDR: Second degree relative
BMI: Body mass index
CVD: Cardiovascular disease
IBD: Inflammatory bowel disease
RCT: Randomised controlled trial
PRISMA: Preferred reporting items for systematic reviews and meta-analyses
CRD: Centre for reviews and dissemination
PROSPERO: International prospective register of systematic reviews
ECG: Electrocardiogram
SES: Socioeconomic status
GP: General practitioner
