# Supplementary material for: Acceptability of predictive testing for ischemic heart disease in those with a family history and the impact of results on behavioural intention and behaviour change: a systematic review
Source: BMC Public Health. 2022 Sep 15;22:1751. doi: 10.1186/s12889-022-14116-6 (PMC9479351; doi:10.1186/s12889-022-14116-6)
Supplement: Supplementary file 1 — Additional file 1. Search strategies from each database used in this review. [file 12889_2022_14116_MOESM1_ESM.pdf]

Additional file 1- Search strategies from each database used in this review

Medline

1	(family adj2 histor*).ti,ab. [mp=title, abstract, original title, name of substance word, subject heading word, floating sub-heading word, keyword heading word, organism supplementary concept word, protocol supplementary concept word, rare disease supplementary concept word, unique identifier, synonyms]
2	"first degree relative".ti,ab.
3	"relative".ti,ab.
4	or/1-3
5	"CVD".ti,ab.
6	exp Heart Diseases/
7	exp Myocardial Ischemia/
8	"ischemic heart disease".ti,ab.
9	ischaemic heart disease.ti,ab.
10	exp Cardiovascular Diseases/
11	Cardiovascular Diseases/px [Psychology]
12	Cardiovascular Diseases/pc [Prevention & Control]
13	exp Coronary Artery Disease/
14	exp Coronary Disease/
15	"coronary heart disease".ti,ab.
16	or/5-15
17	"DNA based test".ti,ab.
18	"gene* screen*".ti,ab.
19	"predict* test".ti,ab.
20	exp Genetic Testing/
21	"genetic risk".ti,ab.
22	Genetic Carrier Screening/
23	(risk adj2 assessment).ti,ab. [mp=title, abstract, original title, name of substance word, subject heading word, floating sub-heading word, keyword heading word, organism supplementary concept word, protocol supplementary concept word, rare disease supplementary concept word, unique identifier, synonyms]
24	(risk adj2 test).ti,ab. [mp=title, abstract, original title, name of substance word, subject heading word, floating sub-heading word, keyword heading word, organism supplementary concept word, protocol supplementary concept word, rare disease supplementary concept word, unique identifier, synonyms]
25	(gene* adj2 test*).ti,ab.

26	(predict* adj2 test*).mp. [mp=title, abstract, original title, name of substance word, subject heading word, floating sub-heading word, keyword heading word, organism supplementary concept word, protocol supplementary concept word, rare disease supplementary concept word, unique identifier, synonyms]
27	exp Genetic Predisposition to Disease/
28	*Genetic Testing/
29	*Risk Factors/
30	*CHOLESTEROL, LDL/
31	*CHOLESTEROL, HDL/
32	*TRIGLYCERIDES/
33	*Lipoproteins/
34	"lipoprotein (a)".ti,ab.
35	"LP (a)".ti,ab.
36	"CRP".ti,ab.
37	or/17-36
38	4 and 16 and 37
39	limit 38 to (humans and “all adult (19 plus years)”)

Embase

1	(family adj2 histor*).ti,ab. [mp=title, abstract, heading word, drug trade name, original title, device manufacturer, drug manufacturer, device trade name, keyword, floating subheading word, candidate term word]
2	"first degree relative".ti,ab.
3	"relative".ti,ab.
4	or/1-3
5	"CVD".ti,ab.
6	exp Heart Diseases/
7	exp Myocardial Ischemia/
8	"ischemic heart disease".ti,ab.
9	ischaemic heart disease.ti,ab.
10	exp Cardiovascular Diseases/
11	Cardiovascular Diseases/pc [Prevention & Control]
12	exp Coronary Artery Disease/

13	exp Coronary Disease/
14	"coronary heart disease".ti,ab.
15	or/5-14
16	"DNA based test".ti,ab.
17	"gene* screen*".ti,ab.
18	"predict* test".ti,ab.
19	exp Genetic Testing/
20	"genetic risk".ti,ab.
21	Genetic Carrier Screening/
22	(risk adj2 assessment).ti,ab. [mp=title, abstract, heading word, drug trade name, original title, device manufacturer, drug manufacturer, device trade name, keyword, floating subheading word, candidate term word]
23	(risk adj2 test).ti,ab. [mp=title, abstract, heading word, drug trade name, original title, device manufacturer, drug manufacturer, device trade name, keyword, floating subheading word, candidate term word]
24	(gene* adj2 test*).ti,ab.
25	(predict* adj2 test*).mp. [mp=title, abstract, heading word, drug trade name, original title, device manufacturer, drug manufacturer, device trade name, keyword, floating subheading word, candidate term word]
26	exp Genetic Predisposition to Disease/
27	*Genetic Testing/
28	*Risk Factors/
29	*CHOLESTEROL, LDL/
30	*CHOLESTEROL, HDL/
31	*TRIGLYCERIDES/
32	*Lipoproteins/
33	"lipoprotein (a)".ti,ab.
34	"LP (a)".ti,ab.
35	"CRP".ti,ab.
36	or/16-35
37	4 and 15 and 36
38	limit 37 to (human and embase and (adult <18 to 64 years> or aged <65+ years>))

Psycinfo

1	(family adj2 histor*).ti,ab. [mp=title, abstract, heading word, table of contents, key concepts, original title, tests & measures]
2	"first degree relative".ti,ab.
3	"relative".ti,ab.
4	or/1-3
5	"CVD".ti,ab.
6	"ischemic heart disease".ti,ab.
7	ischaemic heart disease.ti,ab.
8	"coronary heart disease".ti,ab.
9	or/5-8
10	"DNA based test".ti,ab.
11	"gene* screen*".ti,ab.
12	"predict* test".ti,ab.
13	exp Genetic Testing/
14	"genetic risk".ti,ab.
15	(risk adj2 assessment).ti,ab. [mp=title, abstract, heading word, table of contents, key concepts, original title, tests & measures]
16	(risk adj2 test).ti,ab. [mp=title, abstract, heading word, table of contents, key concepts, original title, tests & measures]
17	(gene* adj2 test*).ti,ab.
18	(predict* adj2 test*).mp. [mp=title, abstract, heading word, table of contents, key concepts, original title, tests & measures]
19	*Genetic Testing/
20	*Risk Factors/
21	*Lipoproteins/
22	"lipoprotein (a)".ti,ab.
23	"LP (a)".ti,ab.
24	"CRP".ti,ab.
25	or/10-24
26	exp Heart Disorders/
27	exp ISCHEMIA/
28	exp Cardiovascular Disorders/
29	coronary artery disease.mp.

30	coronary disease.mp.
31	or/26-30
32	genetic carrier screening.mp.
33	genetic predisposition to disease.mp.
34	*CHOLESTEROL/
35	CHOLESTEROL, LDL.mp.
36	CHOLESTEROL, HDL.mp.
37	triglycerides.mp.
38	or/32-37
39	9 or 31
40	25 or 38
41	4 and 39 and 40
42	limit 41 to (human and adulthood <18+ years>)

LILACS

(("family history" OR "historia familiar" OR "first degree relative" OR "parente de primeiro grau" OR relative OR "parentesco" OR relatives) AND ((ti:"CVD" OR ab:"CVD" OR mh:C14.280* OR mj:"Cardiovascular Diseases" OR mh:"Coronary Artery Disease" OR mh:"Cardiovascular Diseases/psychology" OR mh:"Cardiovascular Diseases/prevention & control") OR ti:("ischemic heart disease" OR "ischaemic heart disease" OR "coronary heart disease") OR ab:("ischemic heart disease" OR "ischaemic heart disease" OR "coronary heart disease")) AND (mh:E01.370.225.562* OR mh:("Genetic Predisposition to Disease" OR "Anticipation, Genetic" OR "Genetic Carrier" OR "Screening Genetic Testing" OR "Risk Factors" OR "CHOLESTEROL, LDL" OR "CHOLESTEROL, HDL" OR "TRIGLYCIDES" OR "Lipoproteins") OR (ti:("DNA based test" OR "predictive test" OR "genetic risk" OR "risk assessment" OR "risk test" OR "lipoprotein (a)" OR "LP (a)" OR "CRP") OR ti:(gene* test*) OR ti:(predict* test*) OR ti:(gene* screen*) OR ab:("DNA based test" OR "predictive test" OR "genetic risk" OR "risk assessment" OR "risk test" OR "predictive test" OR "predictive tests" OR "predictive testing" OR "lipoprotein (a)" OR "LP (a)" OR "CRP") OR ab:(gene* screen*) OR ab:(gene* test*)))) AND NOT (db:"MEDLINE")

----
mh:C14.280* = "Heart Diseases" OR "Myocardial Ischemia" OR "Coronary Disease" but exploded

mh:E01.370.225.562* = "Genetic Testing" but exploded

ProQuest

(AB, TI("family histor*")) OR (AB, TI("first degree relative")) AND (AB, TI("cardiovascular disease*")) OR (AB, TI("ischaemic heart disease")) OR (AB, TI("ischemic heart disease")) AND (AB, TI("gene* test*")) OR (AB, TI("lipoproteins")) OR (AB, TI("triglycerides")).

EThOS

Genetic test AND heart disease AND family history.

Google

(genetic screening) AND (ischemic heart disease) AND (family history).
