# Supplementary material for: Acceptability of predictive testing for ischemic heart disease in those with a family history and the impact of results on behavioural intention and behaviour change: a systematic review
Source: BMC Public Health. 2022 Sep 15;22:1751. doi: 10.1186/s12889-022-14116-6 (PMC9479351; doi:10.1186/s12889-022-14116-6)
Supplement: Supplementary file 2 — Additional file 2. Table of additional information extracted from included studies. [file 12889_2022_14116_MOESM2_ESM.pdf]

Additional file 2- Table of additional information extracted from included studies

References	Ethical Approval	Funding Sources	PPI Involvement
**Claassen et al [57]**	Does not state.	Societal Component of Genomics Research of the Netherlands Organization for Scientific Research (NWO).	No
**Imes et al [58]**	Approved by the University of Washington's institutional review board.	National Institute of Nursing Research of the National Institutes of Health.	No
**Middlemass et al [53]**	Approved by Derby Research Ethics Committee (reference number: 08/H0401/).	National Health Service Task-linked Research and Development funding for 'Clinical Genetics in Primary care' programme.	No
**Stocks et al [56]**	Approved by the Royal Adelaide Hospital Research Ethics committee and Flinders research ethics committee, and approved for conduct at Flinders Private Hospital.	National Health and Medical Research Council (NHMRC) Project.	No
**Saukko et al [59]**	Approval obtained by the Multicentre Research Ethics Committee for Scotland (06/MRE10/9).	UK Department of Health's Genetics Based Health Services Programme.	No
**Sanderson and Michie [55]**	Approved by the Psychology Ethics Board of University College London	MRC-ESRC Postdoctoral Research Fellowship.	No
**Sanderson et al [54]**	Does not state.	Department of Health and Department of Technology, Industry to the London IDEAS Genetics Knowledge Park, Cancer Research UK and the British Heart Foundation.	No
