# Supplementary material for: Acceptability of predictive testing for ischemic heart disease in those with a family history and the impact of results on behavioural intention and behaviour change: a systematic review
Source: BMC Public Health. 2022 Sep 15;22:1751. doi: 10.1186/s12889-022-14116-6 (PMC9479351; doi:10.1186/s12889-022-14116-6)
Supplement: Supplementary file 3 — Additional file 3. Manuals used to guide quality assessment and generate overall scores for quantitative and qualitative studies. [file 12889_2022_14116_MOESM3_ESM.pdf]

Additional file 3- Manuals used to guide quality assessment and generate overall scores for quantitative and qualitative studies

Manual for quantitative studies

Criteria	Definition for quality scoring
Question / objective sufficiently described?	**Yes:** Is easily identified in the introductory section (or first paragraph of methods section). Specifies (where applicable, depending on study design) all of the following: purpose, subjects/target population, and the specific intervention(s) /association(s)/descriptive parameter(s) under investigation. A study purpose that only becomes apparent after studying other parts of the paper is not considered sufficiently described.<br>**Partial:** Vaguely/incompletely reported (e.g. "describe the effect of" or "examine the role of" or "assess opinion on many issues" or "explore the general attitudes"...); or some information has to be gathered from parts of the paper other than the introduction/background/objective section.<br>**No:** Question or objective is not reported, or is incomprehensible.
Study design evident and appropriate?	**Yes:** Design is easily identified and is appropriate to address the study question / objective.<br>**Partial:** Design and /or study question not clearly identified, but gross inappropriateness is not evident; or design is easily identified but only partially addresses the study question.<br>**No:** Design used does not answer study question (e.g., a comparison group is required to answer the study question, but none was used); or design cannot be identified.
Method of subject/comparison group selection or input variables described and appropriate?	**Yes:** Described and appropriate. Selection strategy designed (i.e., consider sampling frame and strategy) to obtain an unbiased sample of the relevant target population or the entire target population of interest (e.g., consecutive patients for clinical trials, population-based random sample for case-control studies or surveys). Where applicable, inclusion/exclusion criteria are described and defined (e.g., "cancer" -- ICD code or equivalent should be provided). Studies of volunteers: methods and setting of recruitment reported. Surveys: sampling frame/ strategy clearly described and appropriate.<br>**Partial:** Selection methods (and inclusion/exclusion criteria, where applicable) are not completely described, but no obvious inappropriateness. Or selection strategy is not ideal (i.e., likely introduced bias) but did not likely seriously distort the results (e.g., telephone survey sampled from listed phone numbers only; hospital based case-control study identified all cases admitted during the study period, but recruited controls admitted during the day/evening only). Any study describing participants only as "volunteers" or "healthy volunteers". Surveys: target population mentioned but sampling strategy unclear.<br>**No:** No information provided. Or obviously inappropriate selection procedures (e.g., inappropriate comparison group if intervention in women is compared to intervention in men). Or presence of selection bias which likely seriously distorted the results (e.g., obvious selection on "exposure" in a case-control study).<br>**N/A:** Descriptive case series/reports.
Subject characteristics sufficiently described?	**Yes:** Sufficient relevant baseline/demographic information clearly characterizing the participants is provided (or reference to previously published baseline data is provided). Where applicable, reproducible criteria used to describe/categorize the participants are clearly defi ned (e.g., ever-smokers, depression scores, systolic blood pressure > 140). If "healthy volunteers" are used, age and sex must be reported (at minimum). Decision analyses: baseline estimates for input variables are clearly specifi ed.

	**Partial:** Poorly defi ned criteria (e.g. "hypertension", "healthy volunteers", "smoking"). Or incomplete relevant baseline / demographic information (e.g., information on likely confounders not reported). Decision analyses: incomplete reporting of baseline estimates for input variables.<br>**No:** No baseline / demographic information provided. Decision analyses: baseline estimates of input variables not given.
If interventional and random allocation was possible, was it described?	**Yes:** True randomization done - requires a description of the method used (e.g., use of random numbers).<br>**Partial:** Randomization mentioned, but method is not (i.e. it may have been possible that randomization was not true).<br>**No:** Random allocation not mentioned although it would have been feasible and appropriate (and was possibly done).<br>**N/A:** Observational analytic studies. Uncontrolled experimental studies. Surveys. Descriptive case series / reports. Decision analyses.
If interventional and blinding of investigators was possible, was it reported?	**Yes:** Blinding reported.<br>**Partial:** Blinding reported but it is not clear who was blinded.<br>**No:** Blinding would have been possible (and was possibly done) but is not reported.<br>**N/A:** Observational analytic studies. Uncontrolled experimental studies. Surveys. Descriptive case series / reports. Decision analyses.
If interventional and blinding of subjects was possible, was it reported?	**Yes:** Blinding reported.<br>**Partial:** Blinding reported but it is not clear who was blinded.<br>**No:** Blinding would have been possible (and was possibly done) but is not reported.<br>**N/A:** Observational analytic studies. Uncontrolled experimental studies. Surveys. Descriptive case series / reports. Decision analyses.
Outcome and (if applicable) exposure measure(s) well defined and robust to measurement / misclassification bias?	**Yes:** Defined (or reference to complete definitions is provided) and measured according to reproducible, "objective" criteria (e.g., death, test completion – yes/no, clinical scores). Little or minimal potential for measurement / misclassification errors. Surveys: clear description (or reference to clear description) of questionnaire/interview content and response options. Decision analyses: sources of uncertainty are defi ned for all input variables.<br>**Partial:** Definition of measures leaves room for subjectivity, or not sure (i.e., not reported in detail, but probably acceptable). Or precise definition(s) are missing, but no evidence or problems in the paper that would lead one to assume major problems. Or instrument/mode of assessment(s) not reported. Or misclassification errors may have occurred, but they did not likely seriously distort the results (e.g., slight difficulty with recall of long-ago events; exposure is measured only at baseline in a long cohort study). Surveys: description of questionnaire/interview content incomplete; response options unclear. Decision analyses: sources of uncertainty are defi ned only for some input variables.<br>**No:** Measures not defined, or are inconsistent throughout the paper. Or measures employ only ill-defi ned, subjective assessments, e.g. "anxiety" or "pain." Or obvious misclassification errors/measurement bias likely seriously distorted the results (e.g., a prospective cohort relies on self-reported outcomes among the "unexposed" but requires clinical assessment of the "exposed"). Surveys: no description of questionnaire/interview content or response options. Decision analyses: sources of uncertainty are not defi ned for input variables.<br>**N/A:** Descriptive case series / reports.
Sample size appropriate?	**Yes:** Seems reasonable with respect to the outcome under study and the study design. When statistically significant results are achieved for major outcomes, appropriate sample size can usually be assumed, unless large standard errors (SE > ½ effect size) and/or problems with multiple testing are evident. Decision analyses: size of modelled cohort / number of iterations specified and justified.<br>**Partial:** Insufficient data to assess sample size (e.g., sample seems "small" and there is no mention of power/sample size/effect size of interest and/or

	variance estimates aren't provided). Or some statistically significant results with standard errors > ½ effect size (i.e., imprecise results). Or some statistically significant results in the absence of variance estimates. Decision analyses: incomplete description or justification of size of modelled cohort / number of iterations.<br>**No:** Obviously inadequate (e.g., statistically non-significant results and standard errors > ½ effect size; or standard deviations > _ of effect size; or statistically non-significant results with no variance estimates and obviously inadequate sample size). Decision analyses: size of modelled cohort / number of iterations not specified.<br>**N/A:** Most surveys (except surveys comparing responses between groups or change over time). Descriptive case series / reports.
Analytic methods described/justified and appropriate?	**Yes:** Analytic methods are described (e.g. "chi square"/ "t-tests"/"Kaplan-Meier with log rank tests", etc.) and appropriate.<br>**Partial:** Analytic methods are not reported and have to be guessed at, but are probably appropriate. Or minor flaws or some tests appropriate, some not (e.g., parametric tests used, but unsure whether appropriate; control group exists but is not used for statistical analysis). Or multiple testing problems not addressed.<br>**No:** Analysis methods not described and cannot be determined. Or obviously inappropriate analysis methods (e.g., chi-square tests for continuous data, SE given where normality is highly unlikely, etc.). Or a study with a descriptive goal / objective is over-analyzed. **N/A:** Descriptive case series / reports.
Some estimate of variance is reported for the main results?	**Yes:** Appropriate variances estimate(s) is/are provided (e.g., range, distribution, confidence intervals, etc.). Decision analyses: sensitivity analysis includes all variables in the model.<br>**Partial:** Undefined "+/-" expressions. Or no specific data given, but insufficient power acknowledged as a problem. Or variance estimates not provided for all main results/outcomes. Or inappropriate variance estimates (e.g., a study examining change over time provides a variance around the parameter of interest at "time 1" or "time 2", but does not provide an estimate of the variance around the difference). Decision analyses: sensitivity analysis is limited, including only some variables in the model.<br>**No:** No information regarding uncertainty of the estimates. Decision analyses: No sensitivity analysis.<br>**N/A:** Descriptive case series / reports. Descriptive surveys collecting information using open-ended questions.
Controlled for confounding?	**Yes:** Randomized study, with comparability of baseline characteristics reported (or non-comparability controlled for in the analysis). Or appropriate control at the design or analysis stage (e.g., matching, subgroup analysis, multivariate models, etc). Decision analyses: dependencies between variables fully accounted for (e.g., joint variables are considered).<br>**Partial:** Incomplete control of confounding. Or control of confounding reportedly done but not completely described. Or randomized study without report of comparability of baseline characteristics. Or confounding not considered, but not likely to have seriously distorted the results. Decision analyses: incomplete consideration of dependencies between variables.<br>**No:** Confounding not considered, and may have seriously distorted the results. Decision analyses: dependencies between variables not considered.<br>**N/A:** Cross-sectional surveys of a single group (i.e., surveys examining change over time or surveys comparing different groups should address the potential for confounding). Descriptive studies. Studies explicitly stating the analysis is strictly descriptive/exploratory in nature.
Results reported in sufficient detail?	**Yes:** Results include major outcomes and all mentioned secondary outcomes.<br>**Partial:** Quantitative results reported only for some outcomes. Or difficult to assess as study question/objective not fully described (and is not made clear in the methods section), but results seem appropriate.

	**No:** Quantitative results are reported for a subsample only, or “n” changes continually across the denominator (e.g., reported proportions do not account for the entire study sample, but are reported only for those with complete data -- i.e., the category of “unknown” is not used where needed). Or results for some major or mentioned secondary outcomes are only qualitatively reported when quantitative reporting would have been possible (e.g., results include vague comments such as “more likely” without quantitative report of actual numbers).
Conclusions supported by the results?	**Yes:** All the conclusions are supported by the data (even if analysis was inappropriate). Conclusions are based on all results relevant to the study question, negative as well as positive ones (e.g., they aren’t based on the sole significant finding while ignoring the negative results). Part of the conclusions may expand beyond the results, if made in addition to rather than instead of those strictly supported by data, and if including indicators of their interpretative nature (e.g., “suggesting,” “possibly”).<br>**Partial:** Some of the major conclusions are supported by the data, some are not. Or speculative interpretations are not indicated as such. Or low (or unreported) response rates call into question the validity of generalizing the results to the target population of interest (i.e., the population defi ned by the sampling frame/strategy).<br>**No:** None or a very small minority of the major conclusions are supported by the data. Or negative findings clearly due to low power are reported as definitive evidence against the alternate hypothesis. Or conclusions are missing. Or extremely low response rates invalidate generalizing the results to the target population of interest (i.e., the population defined by the sampling frame/ strategy).

Manual for qualitative studies

Criteria	Definition for quality scoring
Question / objective sufficiently described?	**Yes:** Research question or objective is clear by the end of the research process (if not at the outset).<br>**Partial:** Research question or objective is vaguely/incompletely reported.<br>**No:** Question or objective is not reported, or is incomprehensible.
Study design evident and appropriate?	**Yes:** Design is easily identified and is appropriate to address the study question.<br>**Partial:** Design is not clearly identified, but gross inappropriateness is not evident; or design is easily identified but a different method would have been more appropriate.<br>**No:** Design used is not appropriate to the study question (e.g. a causal hypothesis is tested using qualitative methods); or design cannot be identified.
Context for the study clear?	**Yes:** The context/setting is adequately described, permitting the reader to relate the findings to other settings.<br>**Partial:** The context/setting is partially described.<br>**No:** The context/setting is not described.
Connection to a theoretical framework / wider body of knowledge?	**Yes:** The theoretical framework/wider body of knowledge informing the study and the methods used is sufficiently described and justified.<br>**Partial:** The theoretical framework/wider body of knowledge is not well described or justified; link to the study methods is not clear.<br>**No:** Theoretical framework/wider body of knowledge is not discussed.
Sampling strategy described, relevant and justified, and includes full range of relevant cases?	**Yes:** The sampling strategy is clearly described and justified. The sample includes the full range of relevant, possible cases/settings (i.e., more than simple convenience sampling), permitting conceptual (rather than statistical) generalizations.<br>**Partial:** The sampling strategy is not completely described, or is not fully justified. Or the sample does not include the full range of relevant, possible cases/settings (i.e., includes a convenience sample only).<br>**No:** Sampling strategy is not described.
Data collection methods clearly described and systematic?	**Yes:** The data collection procedures are systematic, and clearly described, permitting an "audit trail" such that the procedures could be replicated.<br>**Partial:** Data collection procedures are not clearly described; difficult to determine if systematic or replicable.<br>**No:** Data collection procedures are not described.
Data analysis clearly described and systematic?	**Yes:** Systematic analytic methods are clearly described, permitting an "audit trail" such that the procedures could be replicated. The iteration between the data and the explanations for the data (i.e., the theory) is clear – it is apparent how early, simple classifications evolved into more sophisticated coding structures which then evolved into clearly defi ned concepts/explanations for the data). Sufficient data is provided to allow the reader to judge whether the interpretation offered is adequately supported by the data.<br>**Partial:** Analytic methods are not fully described. Or the iterative link between data and theory is not clear.<br>**No:** The analytic methods are not described. Or it is not apparent that a link to theory informs the analysis.
Use of verification procedure(s) to establish credibility?	**Yes:** One or more verification procedures were used to help establish credibility/ trustworthiness of the study (e.g., prolonged engagement in the field, triangulation, peer review or debriefing, negative case analysis, member checks, external audits/inter-rater reliability, "batch" analysis).<br>**No:** Verification procedure(s) not evident.
Conclusions supported by the results?	**Yes:** Sufficient original evidence supports the conclusions. A link to theory informs any claims of generalizability.<br>**Partial:** The conclusions are only partly supported by the data. Or claims of generalizability are not supported.

	**No:** The conclusions are not supported by the data. Or conclusions are absent.
Reflexivity of the account?	**Yes:** The researcher explicitly assessed the likely impact of their own personal characteristics (such as age, sex and professional status) and the methods used on the data obtained.<br>**Partial:** Possible sources of influence on the data obtained were mentioned, but the likely impact of the influence or influences was not discussed.<br>**No:** There is no evidence of reflexivity in the study report.
